# *Withdrawn*: In vitro single‐strand DNA damage and cancer cell cytotoxicity of temozolomide

**DOI:** 10.1002/cam4.786

**Published:** 2019-09-30

Withdrawal: The below listed article has been withdrawn by agreement between the journal’s Editor‐in‐Chief, Qingyi Wei, and John Wiley & Sons Ltd. This action has been agreed because the authors (Purohit S, Jahagirdar D, Kumar A, Sharma NK) withdrew their article in the year 2016–2017 after acceptance, due to non‐payment of APC charges. Unfortunately, a technical error in 2019 led to the article’s publication online. The production team of this journal takes full responsibility and apologizes for the technical error that resulted in the publication of this article. The article “In vitro single‐strand DNA damage and cancer cell cytotoxicity of temozolomide (https://doi.org/10.1002/cam4.786)” published online on September 30, 2019 in Wiley Online Library (http://wileyonlinelibrary.com).

